# Technical note: On the impact of the kV imaging configuration on doses from planar images during motion‐synchronized treatments on Radixact®

**DOI:** 10.1002/acm2.13371

**Published:** 2021-07-26

**Authors:** William S. Ferris, Larry A. DeWerd, John E. Bayouth, Wesley S. Culberson

**Affiliations:** ^1^ Department of Medical Physics School of Medicine and Public Health University of Wisconsin‐Madison Madison Wisconsin USA; ^2^ Department of Human Oncology School of Medicine and Public Health University of Wisconsin‐Madison Madison Wisconsin USA

**Keywords:** kV dose, Radixact, Synchrony, tomotherapy, tracking

## Abstract

Kilovoltage radiographs are acquired during motion‐synchronized treatments on Radixact to localize the tumor during the treatment. Several previous publications have provided estimates of patient dose from these planar radiographs. However, a recent hardware update changed several aspects of the kV imaging system, including a new X‐ray tube, an extended source‐to‐axis distance (SAD), and a larger field size. This is denoted the extended configuration. The purpose of this work was to assess the impact of the configuration change on patient dose from these procedures. Point doses in water were measured using the TG‐61 protocol for tube potentials between 100 and 140 kVp for both the standard and extended configurations under the same water tank setup. Comparisons were made for equal mAs since the same protocols (kVp, mAs) will be used for both configurations. In comparison to the standard configuration, doses per mAs from the extended configuration were found to be ~66% less and falloff less steep due to the increased SAD. However, a larger volume of tissue is irradiated due to the larger field size. Beam quality for a given tube potential was the same as determined by half‐value layer measurements. Both kV configurations are available from the vendor, therefore, the values in this work can be used to compare values previously published in the literature for the standard configuration or to intercompare doses from these two system configurations.

## 1 INTRODUCTION

The Synchrony motion management system on the Radixact linear accelerator (Accuray Inc.) uses kilovoltage (kV) planar radiographs to localize the target during helical tomotherapy treatments.^1^, ^2^, ^3^ The dose the patients receive from these kV radiographs has been investigated in several previous publications.^4^, ^5^ A description of the motion management system can also be found in these publications.

Recently, Accuray has added the capability to acquire kV computed tomography (kVCT) images to the Radixact system. This required several hardware changes, including a new X‐ray tube and detector, addition of a variable collimator, and an extended source‐to‐axis distance (SAD). These changes also apply to the kV radiographs acquired during synchrony treatments, as the same X‐ray tube and detector are used for pre‐treatment 3D kVCT images as for 2D kV radiographs during motion‐synchronized treatments. The kVCT‐enabled version of the Radixact is denoted the “extended configuration” in this work since the major change is the extended SAD of the kV tube, and the version of the Radixact without the ability to acquire kVCT images is denoted the “standard configuration”.

Previous publications on kV radiograph doses from Radixact have only considered the standard configuration.^4^, ^5^ However, both the extended and standard versions of the system are currently available from the vendor and therefore intercomparison of kV radiograph doses is of interest. Therefore, the purpose of this work was to investigate the impact of the changes to the kV imaging configuration on patient dose from kV radiographs during synchronized treatments. The comparison of doses between the two kV configurations of the Radixact will enable clinical users to use values previously published in the literature if they have a different configuration and to intercompare values between two institutions with different configurations.

## 2 METHODS

**FIGURE 1:**
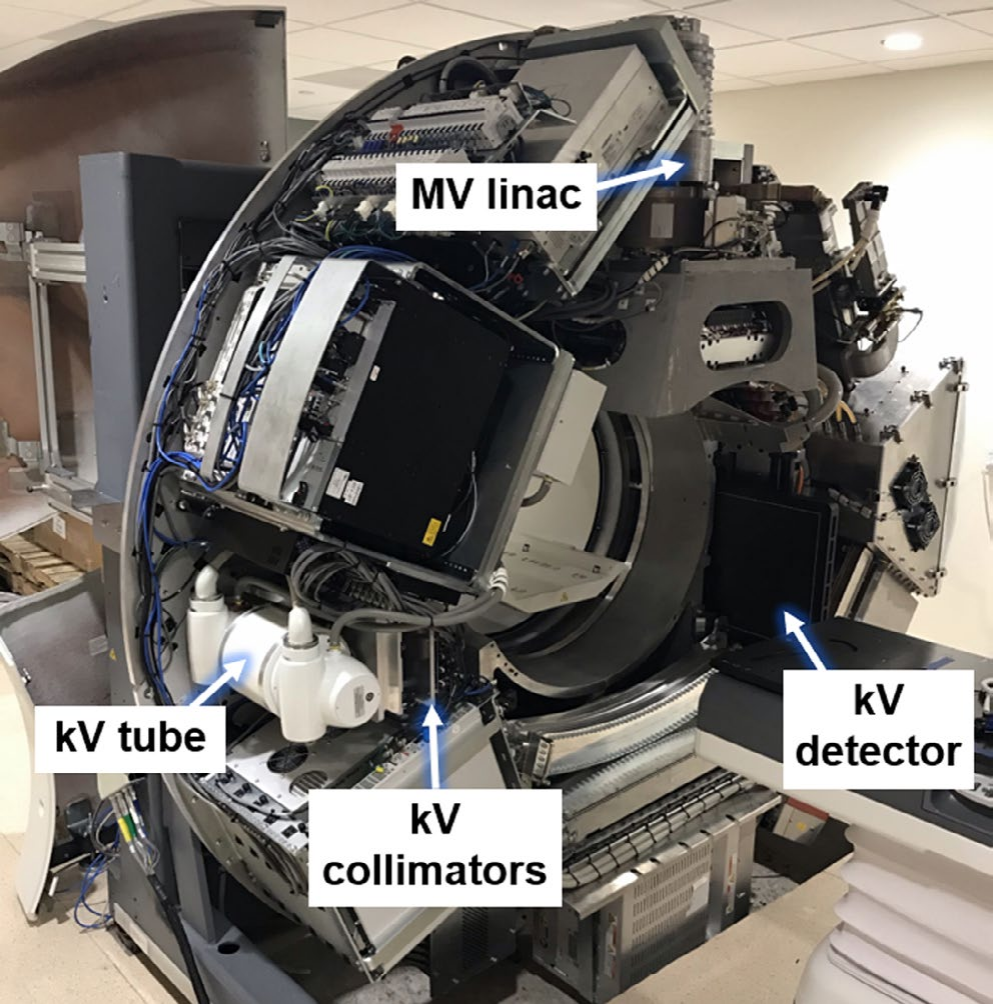
Photograph of a Radixact with the cover removed to show the extended kV imaging configuration. A photograph of the standard imaging configuration is shown in a previous publication^5^

The report of the American Association of Physicists in Medicine (AAPM) Task Group 61 (TG‐61) was used to acquire point dose measurements in water.^6^ Dose to water at the reference depth of 2 cm was calculated using equation 4 in the TG‐61 report.^6^ The measurement geometry of a previous publication on kV dose from Radixact was repeated to isolate differences in point doses due to changing the kV configuration alone.^5^ The same tube potentials were explored: 100, 120, and 140 kVp. Figure 1 shows a photograph of the extended kV imaging configuration on the Radixact. Table 1 is a comparison of several properties of the two configurations. The higher heat capacity and variable collimators are required for the acquisition of kVCT images.

**TABLE 1:** Comparison of properties of the extended and standard kV imaging configurations for radiographs during synchronized treatments. The versions were named in this work based on the SAD as it is the major distinguishing difference

Version	Tube mfr./model	Source‐to‐axis distance (cm)	Field for radiographs at iso (cm × cm)	Anode heat capacity (kJ)
Standard	Siemens SV 150/40/80C	57.5	20 × 20	450
Extended	Varex G‐1592	104.8	28.5 × 23	1100

Half‐value layers (HVLs) in aluminum and copper were measured per TG‐61 for each beam quality using an A12 ionization chamber (Standard Imaging).^6^ Narrow beam geometry was used for HVL measurements and the HVL was obtained using exponential interpolation between nearest measurement points. The HVLs were used to obtain the air‐kerma calibration coefficients (*N_k_*) for the A12 chamber, which was previously calibrated for several X‐ray beams at an Accredited Dosimetry Calibration Laboratory (ADCL).

Point doses in water were measured using an A12 ionization chamber with the kV source at a gantry angle of zero degrees (beam pointing toward the floor). The collimators were set such that the approximate field size at isocenter was 28.5 × 23 cm^2^ (larger transverse than longitudinal), which is the standard field size used for kV radiographs during motion‐synchronized treatments under the extended configuration. The water tank was positioned such that the surface of the water was at an IEC‐Z location of +4.5 cm (towards the source), the same as in previous work.^5^ Point doses were measured along the central axis (IEC‐Z) to produce a depth dose curve, and in the IEC‐X (left/right when head‐first supine) and IEC‐Y (inferior/superior) directions to produce inline and cross‐line profiles. In addition to point doses in water, air kerma at isocenter for the open field was measured with the A12 without a buildup cap to verify the air kerma measurements reported by the manufacturer in the user manual for the extended geometry.

The vendor has indicated that the same protocols will be used as the standard configuration (e.g., Large Thorax protocol is 120 kVp, 1.6 mAs), even with the change in SAD and source‐to‐flat panel detector distance (the flat panel detector for the extended geometry has a higher collection efficiency). Therefore, all measurements in this work were provided per mAs since the same mAs is used between the two configurations. Dose profiles and HVLs were compared for the three energies between the standard and extended configurations.

## 3 RESULTS AND DISCUSSION

**FIGURE 2:**
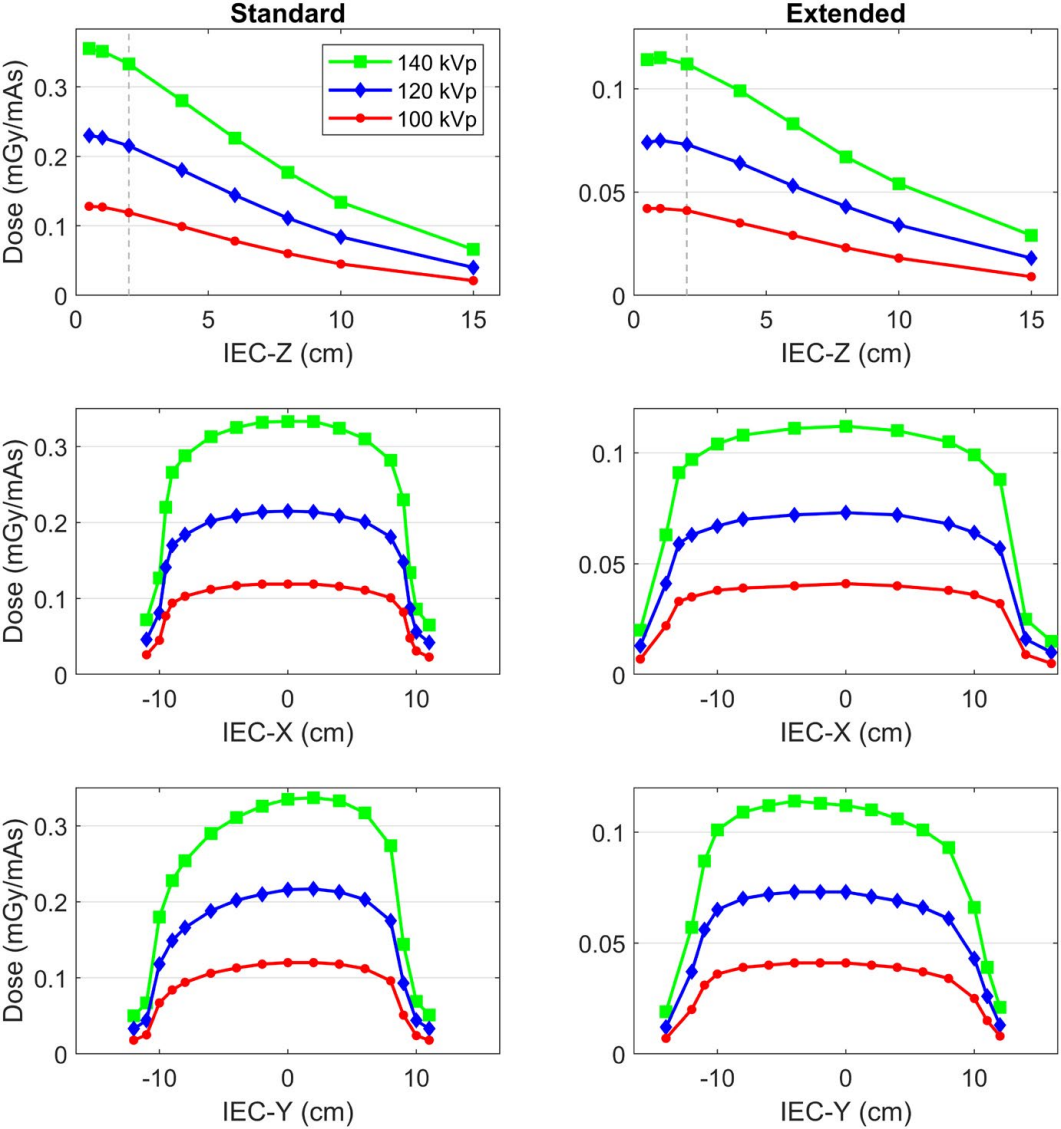
Comparison of point‐dose profiles acquired with an A12 for the standard and extended kV configurations on Radixact. The surface of the water tank is at the same location in the bore for all measurements, at an IEC‐Z of +4.5 cm. The vertical dashed lines indicates the TG‐61 reference depth

Table 2 shows comparisons of several measured properties of the standard and extended kV imaging configurations. Figure 2 shows the measured profiles. Three measurements were acquired and averaged for each point‐dose location. The standard deviation of the three measurements was less than 0.5% for all points. The total uncertainty of each point was estimated to be 5% or less (1σ) based on the measurement and readout uncertainties, and the 4.7% uncertainty for TG‐61 specified doses in water.^6^ The A12 chamber was determined to have a negligible beam quality dependence as the air‐kerma calibration coefficient only varied by 0.7% between HVLs of 4.98 to 14.8 mm Al (UW100M to UW200M).

**TABLE 2:** Comparison of measured properties for the two kV imaging configurations (standard, extended) using an A12 ion chamber and the TG‐61 report^6^

Tube potential (kVp)	Measured HVL (mm Al)	Measured HVL (mm Cu)	*N_k_* (Gy/C)	*D_w_* (mGy/mAs)	Depth‐dose exponential fit^a^
100	9.07, 9.05	0.50, 0.49	4.405E7, 4.405E7	0.119, 0.041	0.084, 0.074
120	10.17, 10.13	0.63, 0.63	4.408E7, 4.408E7	0.215, 0.073	0.078, 0.068
140	11.01, 11.01	0.77, 0.77	4.411E7, 4.411E7	0.333, 0.112	0.074, 0.061

^a^ The value of the parameter *b* in Equation 1.

The reference point dose (at 2 cm depth) of the extended configuration decreased by approximately 66% compared to the standard configuration for equal mAs for all three energies, as observed in Table 2. The inverse square law predicts a 71% decrease based on the differences in the source‐to‐point distances. Beam quality as described by mm Al and mm Cu for a given tube potential was found to be the same. The maximum deviation of HVL was 2% which is within the measurement uncertainty.

The falloff of each depth‐dose curves was compared by fitting the data to the following equation:

(1) $$D=a\cdot \exp\left(‐b\cdot Z\right)+c,$$

where *D* is the dose in mGy/mAs and *Z* is the depth along the central axis. The exponential fit parameter *b* is shown in Table 2 for each curve. Only points at 2 cm depth or deeper were included in the fit. The fit parameter was smaller for all curves with the extended configuration, indicating that the falloff is shallower for the extended configuration due to the decreased inverse‐square dependence.

The air kerma at isocenter was measured to be 0.022, 0.038, and 0.060 mGy/mAs for the 100, 120, and 140 kVp tube potentials, respectively. These values agree with the values reported by the manufacturer in the user manual for the extended geometry. The air kerma at isocenter was determined to be less than the dose at isocenter at 2 cm depth primarily due to the increase in signal from in‐scattered photons when the detector is surrounded by water.

The standard field size for patient radiographs using the extended configuration is 28.5 × 23 cm^2^, which is a 64% increase in field area compared to the 20 × 20 cm^2^ field of the standard configuration. The larger field causes an increased integral dose to the patient (i.e., more tissue irradiated), which can be observed in Figure 2. However, all tissue that is in‐field is expected to receive approximately 66% less dose from the extended configuration. One can also expect a smaller change in field size in the bore with distance away from the source because of the extended SAD. The last notable difference in Figure 2 is that the anode‐cathode axes are both parallel to the direction of table travel, but in opposite direction as indicated by the opposite direction of the heel effect in the Y direction.

The collimators are variable for the extended configuration since this is requirement for the acquisition of kVCT images. It is unclear at this time if users will be able to change collimator settings for kV radiographs during synchronized treatments (such that the field size is different than 28.5 × 23 cm^2^). However, the ability to change collimator settings could be used to decrease integral dose by decreasing the field size, provided that the fiducials or target are still visible in the field of view.

This work did not involve an analysis of image quality versus mAs in the different configurations. The flat‐panel detectors are different with different absorption efficiencies, therefore, caution should be used when trying to assume that similar image quality can be achieved between the configurations by scaling mAs by the values found in this work. However, this work provides the dosimetric framework to be able to optimize patient dose and image quality on each configuration.

## 4 CONCLUSION

The effect of an update to the kV imaging configuration on Radixact on patient dose was explored using HVL measurements and point doses in water. The beam quality as measured by the HVL was determined to be the same for the same tube potential between the standard and extended configurations. Point doses per mAs at 2 cm depth in water for the same tank setup was 66% less for the extended configuration, which is primarily due to the increased SAD. The AAPM Task Group 180 indicates that it is acceptable for uncertainties in imaging doses for radiotherapy patients to reach ±20%.^7^ Therefore, it is acceptable to approximate in‐field doses under the extended configuration by scaling values reported in previous publications for the standard configuration by the decrease in reference dose of 66%.^4^, ^5^

## AUTHOR CONTRIBUTIONS

WSF: Conceptualization, data curation, formal analysis, investigation, methodology, writing ‐ original draft. LAD: Methodology, resources, writing ‐ review and editing. JEB: Conceptualization, supervision, writing ‐ review and editing. WSC: Conceptualization, funding acquisition, resources, supervision, writing ‐ review and editing.

## CONFLICT OF INTEREST

John E. Bayouth has ownership interest in MR Guidance, LCC, which has business activity with a company that utilizes motion management technology (ViewRay, Inc.).
